# Development and application of green and sustainable analytical methods for flavonoid extraction from *Passiflora* waste

**DOI:** 10.1186/s13065-020-00710-5

**Published:** 2020-09-18

**Authors:** Danielle da Silva Francischini, Ana Paula Lopes, Mateus Lodi Segatto, Aylon Matheus Stahl, Vânia Gomes Zuin

**Affiliations:** 1 Department of Chemistry, Federal University of São Carlos, São Carlos, São Paulo 15653-905 Brazil; 2 Green Chemistry Centre of Excellence, University of York, North Yorkshire, YO10 5DD UK; 3 Institute of Sustainable and Environmental Chemistry, Leuphana University, Universitätsallee 1, C13, 13352 Lüneburg, Germany

**Keywords:** Green Analytical Chemistry, green extraction, Sustainable separation, Factorial design, *Passiflora*, Passion fruit, Food chain, Agro-industrial waste, Biorefinery, Flavonoids, UHPLC, Green Star

## Abstract

Brazilian biodiversity and favourable environmental conditions open up possibilities not yet explored, showing potential to shift the country’s monochromatic economy into an emancipated, diversified and sustainable economic environment. This can be made possible through the integral use of its resources, exploring every functional fraction to create novel solutions to modern problems. Biorefineries present an interesting strategy to fully use the potential of agricultural feedstocks and together with green separation methods can contribute to the generation of sustainable processes and products. Passion Fruit (*Passiflora edulis Sims f. flavicarpa* Deg species) is produced on a large scale in Brazil and in other tropical countries, and its processing plants generate tons of residues that basically consist of peel, seeds and bagasse, which account for around 75% of its mass. These fractions of *P. edulis* can contain significant amounts of flavonoids, secondary metabolites that are the main compounds responsible for the fruit’s bioactivity (antioxidant, anti-inflammatory, pesticide and biocide, in general). Therefore, this work aims to develop, apply and compare the best conditions for the extraction of isoorientin, orientin and isovitexin from passion fruit applying solid–liquid methodologies, followed by analyte quantification using UHPLC-PDA. Homogenizer-assisted (HAE), ultrasound-assisted (UAE) and microwave-assisted (MAE) extraction techniques were used, as well as a full factorial design to reach optimal parameters concerning the extraction yield and energy and solvent efficiencies. According to the results, the procedure based on HAE presented the best conditions for the extraction of selected flavonoids (1.07, 0.90 and 0.33 mg g^−1^ of isoorientin, orientin and isovitexin, respectively) and was considered the best method according to the green and sustainable described factors. 
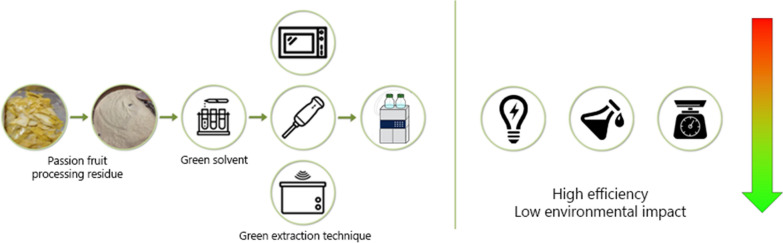

## Introduction

Brazil has a great agricultural potential due to a major biodiversity and favourable environmental conditions that contribute to its agronomic development [1]. According to the Brazilian Institute of Geography and Statistics (IBGE), in 2018 agribusiness activity profits reached R$ 343.5 billion, exceeding the value obtained in 2017 by 8.3% [2]. This raise is directly related to the growth in population and demand for food. However, this demand increases the production and consumption of processed aliments, consequently boosting the amount of waste generated by food processing. The United Nations Food and Agriculture Organization (FAO) estimated that one third of the food produced worldwide is lost or discarded per year [3]. Therefore, it is increasingly necessary to rethink society’s lifestyle and encourage people to adopt processes which use products in their entirety, aiming at a more circular, sustainable future [4, 5].

Considering the processing steps in a food supply chain and reuse of biomass, as well as the principles of Green and Sustainable Chemistry and Green Analytical Chemistry, the biorefinery concept emerges as a sustainable alternative for transforming agro-industrial practices and laboratory activities in order to achieve sustainable processes [1]. In a conceptual waste biorefinery, the residue generated through human consumption, as well as the biomass lost or degraded in the retail and manufacturing stages of food production (bagasse, seeds and peels) is transformed into valuable co-products, which can range from fuels, platform chemicals, nutraceuticals and other high added value compounds [5–7].

Passion fruit is one of the most important fruits in Brazilian agriculture, which reached a production of 600,000 tons in 2018 [2]. The *Passiflora* (*Passifloraceae* family) genus has more than 500 species distributed in Latin America, of which 83 are present in Brazil. *Passiflora edulis Sims f. flavicarpa* Deg (yellow or sour passion fruit) and *Passiflora alata* (sweet passion fruit) are species of economic interest due to a large production of food products worldwide, especially juices and other beverages [8, 9]. Nevertheless, while processing passion fruit, the peel, seeds and bagasse are considered as waste, although it is 75% of the fruit’s mass [10]. This processing residue has significant amounts of oils, carotenoids, proteins, vitamins and phenolic compounds, such as flavonoids, that can be used to produce cosmetics, pharmaceuticals, pesticides and other fine chemical products [8, 11]. Thus, biomass transformation in a biorefinery platform allows an extended use of *Passiflora* fruits and also increases industrial competitiveness among the food supply chain [12, 13].

According to Freire [9], most of the research found in the literature for *Passiflora* aims at studying the chemical composition of its pulp and seed, with scarce studies that explore other parts of the passion fruit that are considered waste, such as the peels. Domínguez-Rodríguez et al. [14] also mentioned the lack of information about the phenolic profile of passion fruit peels and the importance of these samples for the extraction of antioxidant compounds. In the literature, it can also be observed that the focus of most studies is on the analysis of the antioxidant and antibacterial activities of *Passiflora* flavonoids [14, 15] and the nutritional composition of the fruit [16], having the sample preparation a supporting role in these studies.

**Fig. 1:**
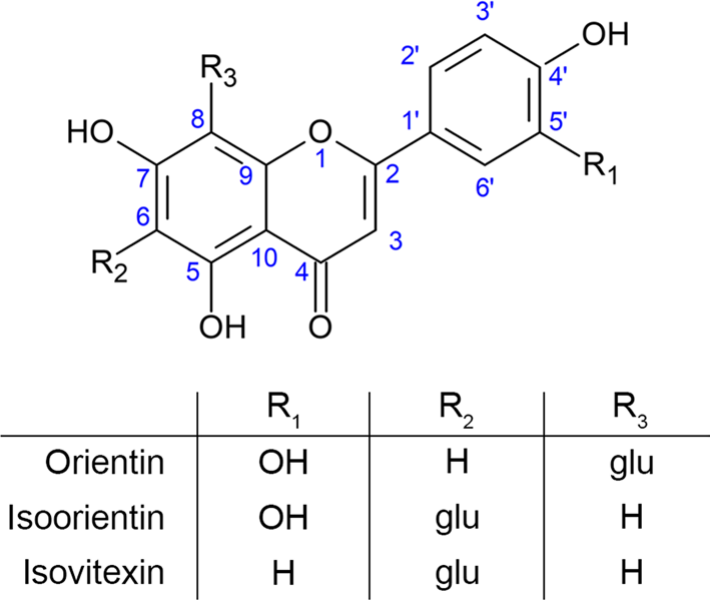
Structure of flavonoids (Adapted from Zeraik [10]); glu, glucose

Flavonoids are compounds that are used to produce food, drugs or cosmetics due to their anti-inflammatory, antioxidant and soothing effects [17, 18]. They are a group of secondary metabolites with a basic molecular structure comprising 15 carbon atoms, represented by a C6–C3–C6 skeleton, in which two aromatic rings are linked through a three-carbon bridge, which is differentiated through substituent molecules (Fig. 1) [19]. Those found in the *Passiflora* species are of the C-glycoside type, which usually contain glucose as substituent molecules directly linked to the aromatic nucleus [17]. The flavonoids isoorientin and orientin can be found in the leaves and pulp of *Passiflora* species [18] and, together with isovitexin, in processing residues of the fruit (peels and seeds) [20–22], are the major compounds responsible for anti-inflammatory [23], antioxidant and antibacterial activities [20].

In the literature, studies that evaluated the efficiency of flavonoid extraction methods in passion fruit can be observed [8, 14, 24, 25], but there is scarcity in mentioning and/or applying Green Analytical Chemistry concepts within this context. Flavonoid extraction based on Green Analytical Chemistry intends to look for alternative and non-toxic solvents, increased energy efficiency and greater extraction yield, as well as reduced operation time, at a lower cost and aiming to mitigate the environmental impacts. Normally, the extraction step corresponds to 40% to 80% of the total cost of chemical processes [26–28]. Different extraction methods have been studied [27], in which solid–liquid extraction methods based on homogenizer-assisted (HAE), ultrasound-assisted (UAE) and microwave-assisted (MAE) extractions are used as sustainable methods to extract bioactive compounds from different types of plant material due to the possibility of using smaller volumes of greener solvents, lower sample mass and safety regarding the analyst [27, 29–31]. The analysis step, among other strategies, can benefit from miniaturization of analytical systems to reduce its environmental impact. This can be seen in Liquid Chromatography systems, one of the most used methods to detect and quantify flavonoids, with the recent development of the Ultra High Performance Liquid Chromatography (UHPLC) technique, which due to a smaller column size and increased pressure can significantly reduce solvent use and analysis time without compromising selectivity and sensibility [32].

Although many analytical methodologies are often considered green and/or sustainable, rarely is there a proper assessment of comparable green and sustainable factors intrinsic to the developed processes. The literature describes different types of greenness assessment metrics that can be used to analyse and compare the green potential of any experiment, mainly comprising of quantitative metrics that measure direct chemical and material efficiency in synthetic chemistry (e.g. atom economy and E-factor) [33]. Recent advances have been reported on metrics specific to analytical chemistry, such as the National Environmental Method Index (NEMI) [34], Analytical Eco-Scale [35] and Green Analytical Procedure Index (GAPI) [36], which mainly focus on laboratory procedures. An adequate metric for sustainable chemistry assessment is the Life Cycle Assessment (LCA), which can evaluate whole systems by quantitative measurements on environmental, economic and social impacts from the material production, manufacturing, use and disposal [33, 37]. The LCA is considered a complex metric because it requires all the inventory data, which may be a very time consuming and impracticable task, thus being replaced by less wholesome metrics [33].

In this study, the biorefinery concept alongside more benign extraction methods could be considered steps towards to more sustainable industrial scale processes but are unaccounted for in those typical green chemistry metrics [7, 38, 39]. By correlating experiment yield (in terms of mass of extracted compounds) with solvent and energy consumption, data can be obtained to analyse efficiency and sustainability aspects. This is an important approach to compare different extraction techniques and their scalability, which is a simpler and more holistic appraisal.

Therefore, the present work sought to integrate the biorefinery concepts when establishing methods for extracting bioactive compounds from *Passiflora edulis fo. flavicarpa* (yellow passion fruit) peel, from a conventional farming model considering the principles of Green Analytical Chemistry [40]. Methods based on renewable solvents to extract orientin, isoorientin, isovitexin were developed, optimised and applied, as well as the development of the analytical method to detect and quantify these compounds using a UHPLC-PDA-QDa system. To evaluate the green and sustainable aspects, different comparison approaches were adopted to estimate energy and solvent volume efficiency for each extraction method.

## Methods

### Sample conditioning

The passion fruit samples were acquired from the city of São Carlos, São Paulo state, Brazil, in a local market. The collection and handling of the plant material were performed with compliance with institutional and national guidelines, including the register at the Brazilian National Management System of the Genetic Patrimony and Associated Traditional Knowledge (SisGen), register code A4C7EC8. Approximately 30 pieces of fruit were peeled, separating the yellow shell (mesocarp) from the white interior (epicarp). The yellow peels were dried in an air circulation oven for 7 days at 35 °C, initially blended in a domestic blender, followed by milling in an analytical mill and sieved to obtain particle sizes smaller than 60 mesh.

### Chemicals and reagents

The analytical standards of the flavonoids isoorientin and orientin were purchased from Sigma-Aldrich^®^ (assay ≥ 95%, São Paulo, SP, Brazil) and isovitexin from HWI Pharma Solutions (assay ≥ 90.38%, Rülzheim, Germany). For the extraction of flavonoids in the passion fruit peel, ethanol (HPLC grade, Panreac^®^, Barcelona, Spain) and distilled water were used. In the chromatographic separation, acetonitrile (HPLC grade from Tedia, Fairfield, US), methanol (HPLC grade from Honeywell, Charlotte, North Caroline, US), ultrapure water (Mili-Q system from Millipore^®^, Burlington, US) and formic acid (Sigma-Aldrich, São Paulo, Brazil, assay ≥ 95%) were used.

### Extraction techniques and factorial design

Factorial designs were applied for each extraction technique. Table 1 summarizes the variables and their levels which were studied for HAE, UAE and MAE experiments, as can be seen below. Details for each technique are found in the following topics.

**Table 1:** Experimental variables for the different extraction methods studied

Extraction method	Variables	Unit	Levels		
			− 1	0	1
HAE	Sample/Solvent ratio	–	0.05	–	0.1
	EtOH/H_2_O	%	30	–	70
	Time	Min	2	–	8
UAE	Sample/Solvent ratio	–	0.05	0.07	0.1
	EtOH/H_2_O	%	30	50	70
	Time	Min	15	38	60
MAE	Sample/Solvent ratio	–	0.05	0.07	0.1
	EtOH/H_2_O	%	30	40	50
	Time	Min	5	15	25
	Temperature	°C	60	90	120

### Homogenizer assisted extraction (HAE)

The HAE extraction was performed by IKA’s T10 basic Ultra-Turrax^®^ (Staufen im Breisgau, Germany) mixer. The dried sample was weighed with an analytical balance and transferred to a 15 mL falcon tube, followed by the addition of 5 mL of the solvent and extraction using the mixer at a fixed rotation of 15.360 rpm for a variable time, according to the factorial design. A complete factorial design 2^3^ was developed in which the variables’ sample/solvent ratio, time and concentration of ethanol in water were evaluated, thus resulting in 8 experiments. In order to evaluate the relative standard deviation (SD) and the reproducibility of the extraction method, replicates were performed in three different experiments.

### Ultrasound assisted extraction (UAE)

The UAE was performed using Ultrasonic Cleaning, Soni-Tech TDRFORCE^®^ (São Bernardo dos Campos, Brazil) equipment with fixed power at 125 W. The samples were weighed, according to the sample/solvent ratio, and added to a 25 mL glass tube with a fixed volume of 5 mL of the solvent, which was placed in the ultrasonic bath and followed by extraction. A complete factorial design 2^3^ with a central point was applied resulting in 9 experiments. The central point experiment was performed in triplicate in order to assess the relative standard deviation and the reproducibility of the extraction method. The evaluated variables were sample/solvent ratio, time and concentration of ethanol in water.

### Microwave assisted extraction (MAE)

The MAE extraction method was performed by Berghof-Speedwave^®^ (Baden-Württemberg, Germany) equipment, with a fixed power of 800 W and 30 bars of maximum pressure. The procedure started by weighing the dried samples on an analytical balance and adding it to the microwave flask with 7 mL of the solvent, which was placed inside the microwave oven. The heating started with a ramp from 30 °C to the desired temperature, which remained constant for the time selected for each experiment. To carry out the experiments, a 24 experimental design with a central point was developed, thus resulting in 17 experiments, considering the central point to be performed in quadruplicate in order to assess the relative standard deviation and the reproducibility of the method. The variables studied were the sample/solvent ratio, time, temperature and concentration of ethanol in water.

### Multivariable analysis

In order to assess the importance of the variables chosen in the studied extraction methods, complete factorial designs were used for each experiment (please check Additional file 1: Tables S1–S3). Thus, the influence of each variable (primary effects) and the influence of the interaction between variables (secondary effects) were calculated and the effect graph was obtained. The values obtained in concentration per mass of sample (mg g^−1^) of each flavonoid were multiplied by the coded value of the factorial design (− 1, 0 or + 1) for each variable analysed and summed up, obtaining the effect values (E). Thus, the ratio between the squared effect (E^2^) values and the sum of the squared effects represents the percentage that each variable influences the extraction yield, which can be found in Additional file 2 for all three techniques.

### Analysis and quantification of flavonoids by UHPLC-PDA

The quantitative analysis of the flavonoids was performed using the Ultra High Performance Liquid Chromatography (UHPLC) system Waters ACQUITY H-class UPLC^®^ (Milford, US), coupled with Photodiode Array UV (PDA) and QDa mass spectrometer detectors, in which the instrumental parameters as the composition of mobile phase and the composition gradient, the column, flow rate and temperature were previously optimised in order to obtained analytical signal with high resolution. The mobile phase consisted of ultrapure water acidified with 0.1% formic acid (A) and acetonitrile (B). The extracts were centrifuged at 20 °C (10,000 rpm) for 20 min and the supernatant was filtered through a 20 μm PTFE membrane, followed by an injection of 1 μL of the resulting solution. The stationary phase used was the ACQUITY HSS C18 SB column (Waters, Milford, MA, US, 1.8 μm; 2.1 × 100 mm) at 40 °C. The elution occurred in a gradient mode with a flow rate of 0.3 mL.min^−1^, starting with 10% of B and reaching 100% of B in a 25-min gradient. The chromatograms were recorded in a fixed wavelength of 330 nm. For each flavonoid, an analytical curve was prepared in methanol in five different concentrations: 10, 20, 40, 60, 80 and 100 mg L^−1^ and stored at 4^◦^C.

### Figures of merit

To carry out the validation of the analytical method, the parameters Specificity, Linearity, Recovery, Precision and the Limits of Detection (LOD) and Quantification (LOQ) were evaluated in accordance with the International Council for Harmonization (ICH) standard [41].

To assess specificity, the UV–Vis and mass spectra of each flavonoid obtained at three different retention times at a wavelength of 350 nm were compared. For orientin, the retention times were 5.097, 5.114 and 5.048 min, for isoorientin 5.309, 5.292 and 5.424 min and for isovitexin 6.365, 6.408 and 6.333 min. The linearity was studied from the correlation coefficient (R^2^ > 0.99), and the values of LOD and LOQ were calculated from the analytical curve, according to the ICH guidelines. To evaluate the steps of recovery and precision (intra-day) three extractions of passion fruit sample were carried out with 400 µL of standard solution in different concentrations, obtaining the final concentrations of 10, 20, 40 mg L^−1^ of orientin, isoorientin and isovitexin.

### Green and sustainable factors

Besides the concentration per mass of sample, three other parameters were calculated in order to evaluate green and sustainable factors and industrial feasibility for all extraction methods: sample mass, energy and solvent volume efficiency. These were obtained based on the final mass of the analytes for each experiment by calculating the amount of each parameter consumed per mass of analyte extracted. The energy spent (watt-hour) on each experiment was determined by multiplying the equipment’s power (watts) by the total extraction time (hours), while sample mass and solvent volume were determined by the factorial design. Each efficiency was calculated by dividing the values of energy (kWh), sample mass (kg) and solvent consumption (L) by the value of mass (in g) of the analyte for each experiment.

## Results and discussion

### Calibration curve and figures of merit

The parameters regarding the validation of the calibration curve showed an acceptable correlation coefficient (R^2^ > 0.99), as well as LOD and LOQ consistent with the extraction yields. Table 2 summarizes the validation data for each analyte. Recovery essays in the best extraction technique resulted in values from 89.5 to 99.35%, which are inside the acceptable values for most validation guidelines (80–120%). Intra-day error, as represented by the variation coefficient, showed errors from 0.22 to 3.86%, a good range that represents low fluctuations in the peak area values within the same day of analysis. Specificity was evaluated using the UV-profile in three retention times within a peak and QDa m/z values for each peak in extracts from all extraction techniques. The UV and QDa profiles of standards compounds and their related peaks in the extracts can be seen in Additional file 3, and the chromatograms for each extraction method are presented in the next sections. Acceptable peak purity can be observed for the proposed analytes considering both criteria.

**Table 2:** Calibration curves for orientin, isoorientin and isovitexin and linearity parameters

Compound	Equation	R^2^	LOD (mg L^−1^)	LOQ (mg L^−1^)	Recovery (%)	Intra-day error (%)
Orientin	y = 6637.2x − 45232	0.9951	4.28	14.27	97.40–99.35	0.22–2.15
Isoorientin	y = 8188.1x + 13382	0.9995	2.00	6.65	96.49–99.35	0.34–1.52
Isovitexin	y = 8210.9x + 22235	0.9994	0.90	2.90	89.50–95.72	1058–3.86

### Homogenizer assisted extraction (HAE)

**Fig. 2:**
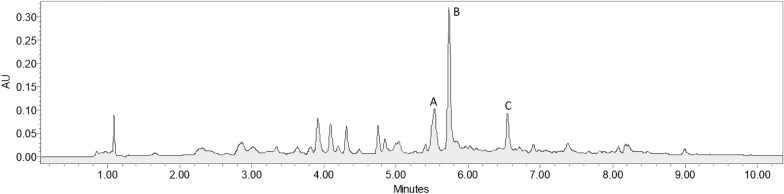
Chromatography profile of P. edulis alcoholic extract, obtained from HAE, with the following analytes: (**a**) orientin, (**b**) isoorientin and (**c**) isovitexin

Following the analytical procedure and chromatographic method describe in session 2.4, the chromatography profile of *P. edulis* extract was obtained, as can been seen in Fig. 2, in which each analyte could be detected and efficiently separated. The experimental design described for each extraction methodology were used and the responses were obtained using the extraction yield in mg of analyte per gram of sample. The results for extraction using the HAE method, as well as its factorial design, are shown in Additional file 1: S1. The conditions used in experiment 8 (all variables in maximum level, + 1) showed the best yield for all analytes, with isoorientin at a higher concentration than the other analytes (1.11 mg g^−1^).

**Fig. 3:**
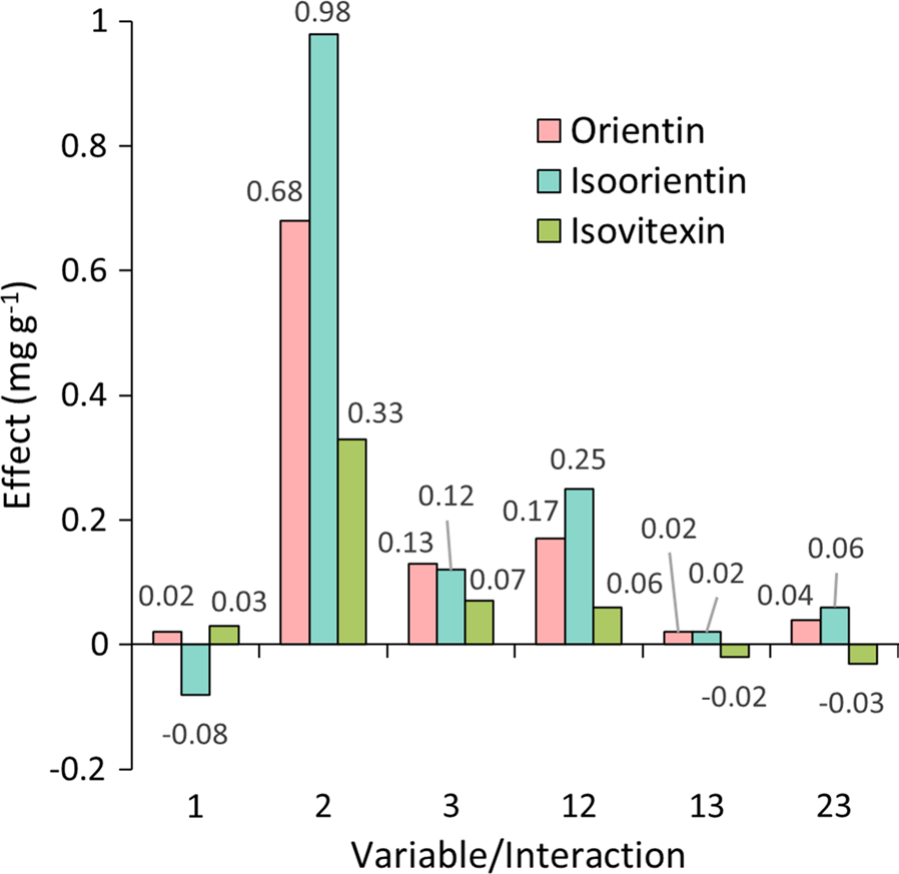
Effect of each variable and their interactions on the responses of HAE methodology

Multivariate analysis was carried out by calculating the influence of each variable (primary effects) and the influence of the interaction between variables (secondary effects) in the final response. Figure 3 shows the calculated effects in mg g^−1^ for the extractions with HAE, as ruled by the variables 1 (sample/solvent ratio), 2 (ethanol/water concentration) and 3 (extraction time). For all three analytes, variable 2 had a major positive influence on the final response, accounting for 92% of the effects for isoorientin and 91% for both orientin and isovitexin. This means that a higher percentage of ethanol in water was mainly responsible for improving the extraction yield for all analysed compounds, considering the range selected for HAE (30–70% of EtOH/H_2_O). This can be seen in the responses from experiments 3, 4 and 7 (Additional file 1: S1), in which the variations between their responses and the experiment with the highest yield (8) are less than 10%. Most of the other variables and their interactions showed slightly positive effects, although low percentual values, meaning that their influence is small or not significant to the response. Such observations can lead us to conclude that the solubility change when varying the percentage of ethanol in water has a higher effect than the other parameters studied in this extraction technique, proving to be a key aspect regarding HAE methodologies. In this case, all three analytes have shown better extraction yields in a 70% solution of ethanol in water.

### Ultrasound assisted extraction (UAE)

**Fig. 4:**
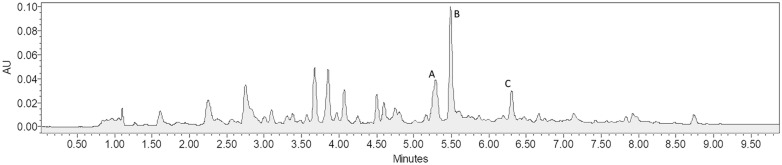
Chromatography profile of P. edulis alcoholic extract, obtained from UAE, with the following analytes: (**a**) orientin, (**b**) isoorientin and (**c**) isovitexin

The chromatography profile of *P. edulis* extract can be seen in the Fig. 4, in which is possible to observe that the signal intensity of the analytes in UAE method were lower compared to HAE extraction. Results from UAE (Additional file 1: S2) showed similar extraction yields for orientin and isoorientin, which both peaked in experiments 1 and 5, although at lower levels than in HAE experiments. Isovitexin has also been found in lower levels, reaching up to 0.17 and 0.18 mg g^−1^ in experiments 1 and 5, respectively.

**Fig. 5:**
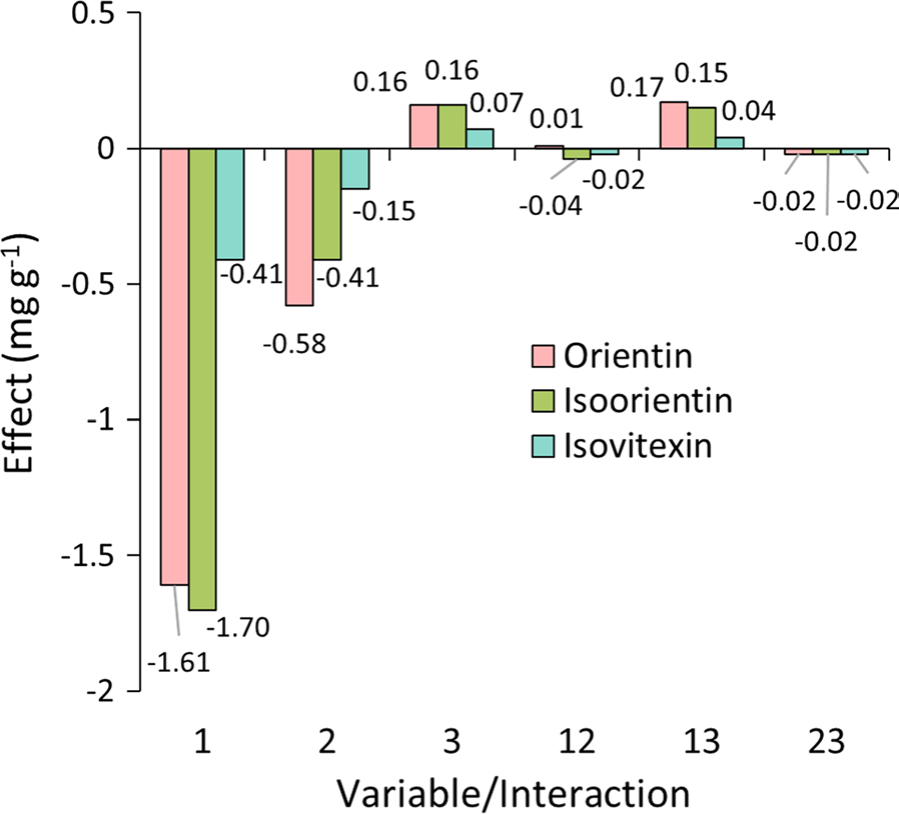
Effect of each variable and their interactions on the responses of UAE methodology

According to the analysis of the effects of each parameter in UAE (Fig. 5), variables 1 (sample/solvent ratio) and 2 (concentration of ethanol in water) had higher negative effects on the response for all three compounds. Sample/solvent ratio accounted for 87%, 93% and 86% of the total effect for orientin, isoorientin and isovitexin, respectively. Due to a negative effect, a higher response is found when a smaller sample/solvent ratio is used, i.e. a higher volume of solvent per mass of sample. Ethanol concentration in water, although showing effects no higher than 11%, negatively affected the response, which means that a smaller  %EtOH/H_2_O is preferable in this condition. Experiments with higher extraction yields (1 and 5) were performed with both variables 1 and 2 at lower levels, although experiment 1 may be preferable due to a lower time, and therefore a lower energy consumption.

### Microwave assisted extraction (MAE)

**Fig. 6:**
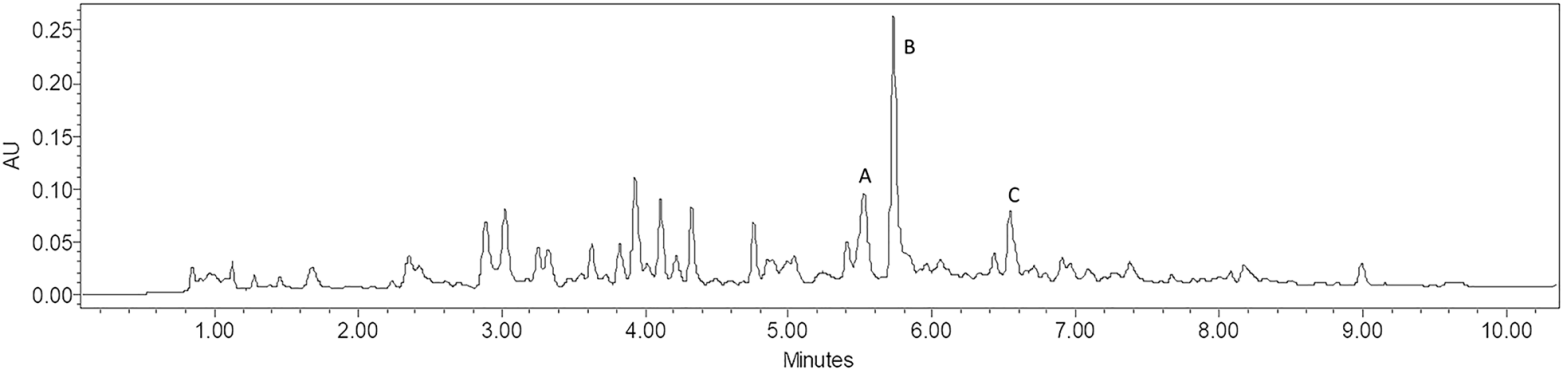
Chromatography profile of P. edulis alcoholic extract, obtained from MAE, with the following analytes: (**a**) orientin, (**b**) isoorientin and (**c**) isovitexin

The Fig. 6 presents the chromatography profile of *P. edulis* extract and can be seen that the analytical signal of all three analytes were similar compared to HAE, although with slightly lower yields (Additional file 1: S3). Higher concentrations of orientin were obtained in experiments 17 (0.89 mg g^−1^), 9 (0.88 mg g^−1^) and experiments 10 and 2 (0.82 mg g^−1^). Experiment 10 was the most successful in terms of extracting isoorientin (0.94 mg g^−1^) and isovitexin (0.34 mg g^−1^), followed by experiments 5, 17 and 9, showing a more complex influence of the variables in the final results, as will be discussed below.

**Fig. 7:**
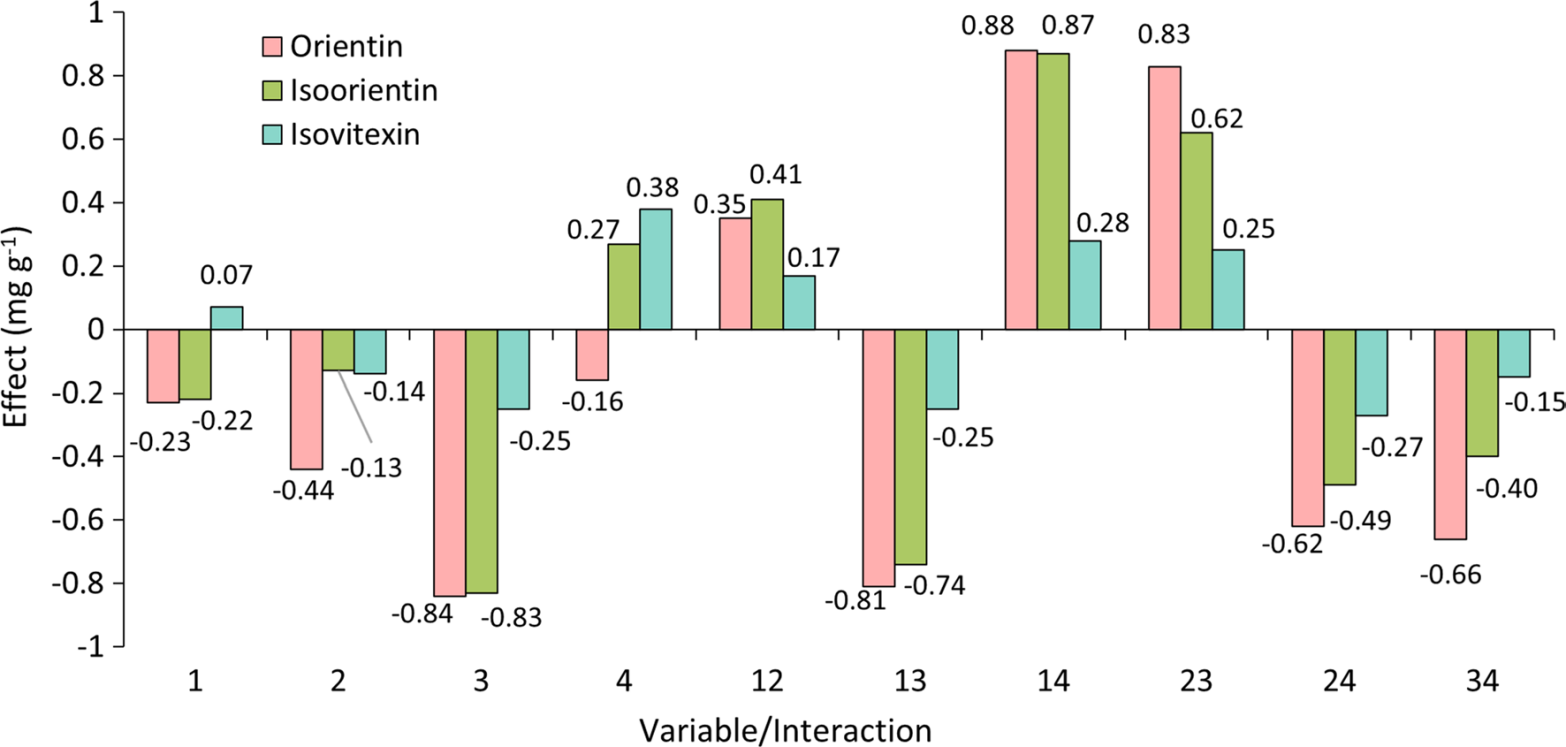
Effect of each variable and their interactions on the responses of MAE methodology

It can be observed in Fig. 7 that the interaction between variables 1 (sample solvent) and 4 (temperature) had a major positive effect on the extraction of orientin and isoorientin, with an influence of 19.2 and 24.5%, respectively. This means that when both variables were at a maximum level, a higher extraction yield was observed. This is closely followed by the effects of variable 3 (extraction time), which had a negative impact of 17.5% for orientin and 22.3% for isoorientin, meaning that higher responses were obtained from the experiments with variable 3 in the lower level. Isovitexin, on the other hand, was mostly influenced by variable 4 (temperature), responsible for 26.1% (positively) of the total effect for this analyte. Therefore, experiments 17 (all variables in central point) and 10 (variables 1 and 4 in higher levels and variables 2 and 3 in lower levels) showed the highest extraction yields, in which experiment 10 yielded slightly better for isoorientin and isovitexin.

### Comparative assessment

#### Extraction yield

To compare the extraction methods, experiment 8 from HAE, experiment 1 from UAE and experiment 10 from MAE were selected due to their optimal yield, in terms of concentration of analyte per mass of sample, as can be seen in Table 3. HAE presented the best results for flavonoid concentration. Comparing it to MAE, orientin and isoorientin values are 12.7% and 15.3% higher, respectively, and isovitexin extraction yields were equal. Moreover, the homogenizer-assisted technique presented higher concentrations than UAE, which were 25.5%, 38.7% and 50% higher for orientin, isoorientin and isovitexin, respectively.

**Table 3:** Comparison between experimental yields of each extraction methods

Extraction method	Experimental yield		
	mg g^−1^		
	Orientin	Isoorientin	Isovitexin
HAE	0.94	1.11	0.34
UAE	0.70	0.68	0.17
MAE	0.82	0.94	0.34

#### Green and sustainable factors

In order to evaluate green and sustainable factors and estimate the possible scalability, the amount of sample (kg g^−1^), energy (kWh g^−1^) and solvent (L g^−1^) required to extract 1 g of each analyte was calculated. For each extraction method, experiments that show a variation of up to 10% in relation to the highest extraction yield (mg g^−1^ of sample) were compared. The results for all selected experiments can be seen in Additional file 4.

For the HAE method, experiments 8, 3, 4 and 7 were selected for analysis. When comparing the results obtained, it can be observed that experiment 4 presents the best values for sustainable factors and requires, on average, 74% less energy when compared to experiment 8, although the latter demands 4% less solvent and slightly less sample mass to extract 1 g of each analyte (3%). In the UAE method, experiments 1 and 5 were selected, and the calculated factors show that experiment 1 requires on average 74% less energy than experiment 5, but 3% more solvent volume and sample mass. Regarding the MAE methodology, experiment 10 and 17 were selected, and from the results, it can be noted that experiment 10 requires 77% less energy, 33% less solvent volume and 6% less sample mass to extract 1 g of the analytes, on average.

**Fig. 8:**
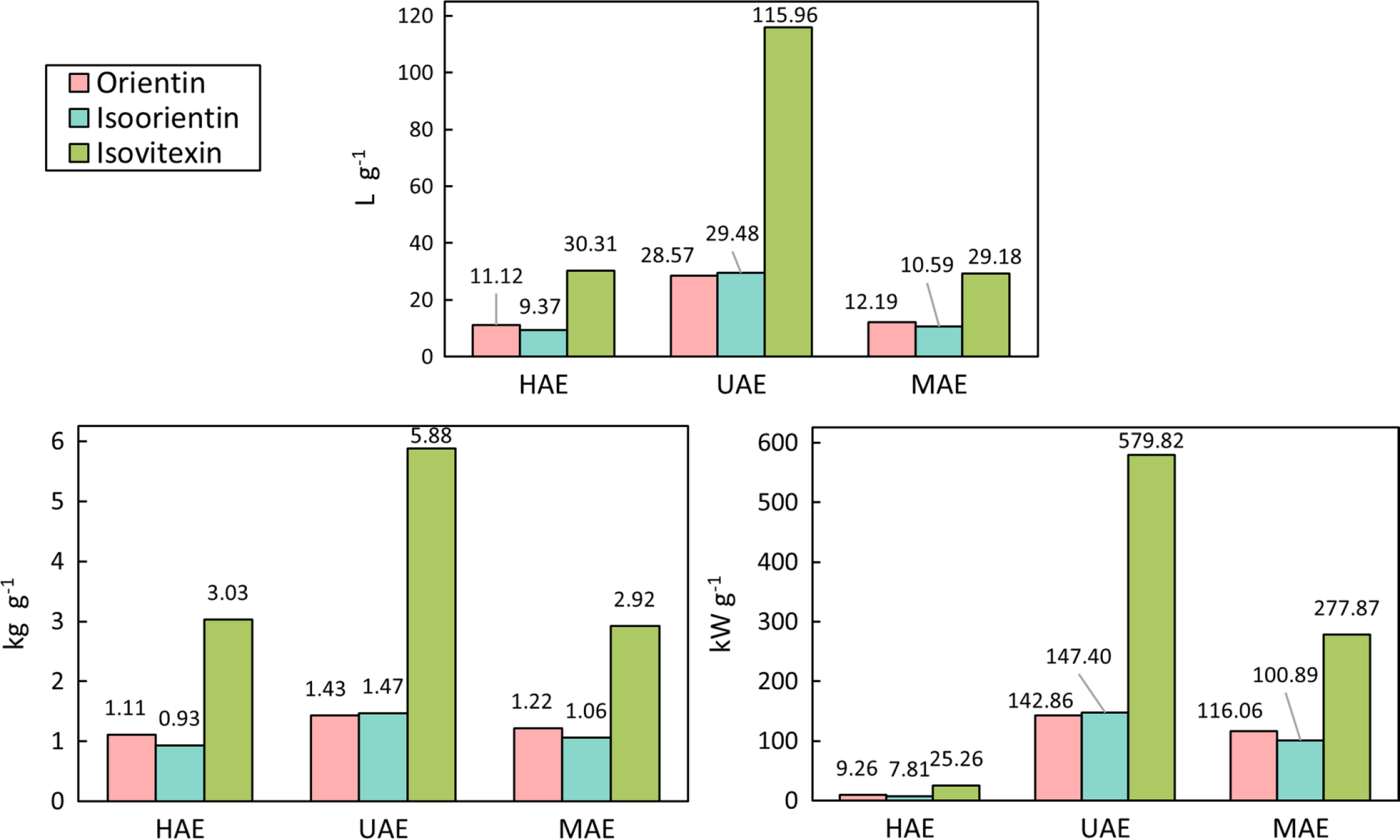
Comparison between methods for sample mass, solvent volume and energy consumption efficiencies

As can be seen in Fig. 8, the comparison of three methods concerning sustainable factors also lead to HAE (experiment 4) being the most environmentally safe among the techniques, consuming approximately 91% and 95% less energy on average than MAE (experiment 10) and UAE (experiment 1), respectively. Furthermore, the Homogenizer-based method had an average of 71% less solvent consumption than UAE and 3% less than MAE, while keeping a good ratio of extraction per mass of sample, as discussed in Sect. “Extraction yield”.

## Discussion

The HAE has been described in some works due to its low cost, time and energy consumption for phenolic compound extraction from plant materials such as banana passion fruit [42], honeysuckle flowers [43], seeds and other plant materials of berry fruit [44]. The mechanism behind the HAE method involves solvent diffusion into the cell through mechanical rupture caused by high-performance agitation, extracting the compounds [43]. In UAE, the extracting mechanism occurs by interaction between the cell wall and ultrasound waves, which promotes cell rupture and solvent diffusion. The extracting phenomenon for MAE is similar to UAE, except it applies thermic waves instead of ultrasounds waves. The difference between MAE and other heating mechanisms are the heat exchange directions, which also occurs from the inside of cells to the outside due to the microwave radiation, enhancing cell rupture and the release of the analytes [30, 45, 46]. MAE and UAE techniques are promising alternatives for flavonoid study in plant matrixes such as passion fruit. This is supported by the comparable results of these extraction methods with HAE. However, according to Vinatoru et al. and Zhang et al. [30, 47], due to their sound or thermic waves-based phenomenon, degradation of phenolic compounds may occur with long sonication time or high microwave temperatures, thus decreasing the extraction yield.

It is important to observe that assessing factors related to sustainability and scalability in lab scale extraction methodologies, as energy and solvent volume efficiency, could be essential to help evaluate best parameters and comparing as well applying different techniques [48, 49]. The case of experiments 4 and 8 from HAE, for example, showed that although very comparable extraction yields (mg.g^−1^)—which is usually the most assessed response in extractions of natural products—experiment 4 was chosen to be superior due to a higher energy efficiency. An analogous conclusion was observed in the comparison between HAE and MAE selected experiments, which had similar performances considering the concentration and solvent volume efficiency, but MAE showed to require more energy than HAE in this study. Such factors can help us distinguish efficiency in terms of mass yield and in terms of energy and solvent consumption, helping us to better understand true sustainability for each technique and to make a fairer comparison of green technologies. In this case, we concluded that two extraction techniques (MAE and UAE) that are usually listed as green and/or sustainable methods, had lower energy and solvent efficiencies than HAE, which is usually neglected as a sustainable technology for the extraction of natural products.

## Conclusion

The integration of biorefinery concepts and green analytical techniques were investigated in this case study. By applying more benign extraction techniques such as HAE, UAE, MAE, using renewable solvents and performing a factorial design, more sustainable separation methods were developed, optimised and applied aiming at obtaining value-added bioactive compounds such as orientin, isoorientin and isovitexin from Brazilian passion fruit waste. The comparison of the quantitative results obtained by UHPLC-PDA showed HAE as the one with better extraction yields and lowest energy consumption in optimum parameters of 0.1 sample/solvent ratio, 70% solution of ethanol in water and 2 min of extraction. Presenting the best extraction yield, energy and solvent efficiencies, HAE has shown to be the greenest and most efficient method among the studied methods. The combination of these findings can help us to understand the different extraction techniques and their relevant variables, as well as taking a further step into a feasible passion fruit waste sustainable biorefinery, by using scalable processes, boosting circular economy in the food supply chain.

## Supplementary information

**Additional file 1:** Normalized effects for variables from all three extraction techniques.

**Additional file 2:** UV profile and QDA data of extracts from all three extraction techniques.

**Additional file 3:** Experimental design and responses for orientin, isoorientin and isovitexin from all three extraction techniques.

**Additional file 4:** Results of green and sustainable factors from all three extraction techniques.

## Data Availability

The datasets supporting the conclusions of this article are included within the article and its Additional files 1, 2, 3, 4.

## Abbreviations

*P. edulis*: *Passiflora edulis*
HAE: Homogenizer-assisted extraction
UAE: Ultrasound-assisted extraction
MAE: Microwave-assisted extraction
IBGE: Brazilian Institute of Geography and Statistics
FAO: Food and Agriculture Organization
UHPLC: Ultra High Performance Liquid Chromatography
PDA: Photodiode Array
QDa: Mass spectrometer detectors
UV: Ultraviolet radiation
NEMI: National Environmental Method Index
GAPI: Green Analytical Procedure Index
LCA: Life Cycle Assessment
SD: Standard deviation
E: Squared effects
LOD: Limits of detection
LOQ: Limits of quantification
ICH: International Council for Harmonization
R: Correlation coefficient
EtOH: Ethanol
H_2_O: Water
