# Supplementary material for: Development and application of green and sustainable analytical methods for flavonoid extraction from Passiflora waste
Source: BMC Chem. 2020 Sep 18;14(1):56. doi: 10.1186/s13065-020-00710-5 (PMC7501698; doi:10.1186/s13065-020-00710-5)
Supplement: Supplementary file 1 — Additional file 1: Normalized effects for variables from all three extraction techniques. [file 13065_2020_710_MOESM1_ESM.docx]

**Table S1:** Experimental design and responses for orientin, isoorientin and isovitexin using HAE method.

| **Experiment** | **Sample/ Solvent ratio** | **EtOH/H_2_O (%)** | **Time** | **Orientin** | **Isoorientin** | **Isovitexin** |
| --- | --- | --- | --- | --- | --- | --- |
|  |  |  |  | mg g^-1^ | | |
| **1** | -1 | -1 | -1 | 0.74 | 0.86 | 0.23 |
| **2*** | 1 | -1 | -1 | 0.69 | 0.75 | 0.23 |
| **3** | -1 | 1 | -1 | 0.85 | 1.03 | 0.31 |
| **4*** | 1 | 1 | -1 | 0.90 | 1.07 | 0.33 |
| **5*** | -1 | -1 | 1 | 0.75 | 0.87 | 0.27 |
| **6** | 1 | -1 | 1 | 0.73 | 0.79 | 0.25 |
| **7** | -1 | 1 | 1 | 0.90 | 1.07 | 0.32 |
| **8** | 1 | 1 | 1 | 0.94 | 1.11 | 0.34 |

*triplicate

**Table S2:** Experimental design and responses for orientin, isoorientin and isovitexin using UAE method.

| **Experiment** | **Sample/ Solvent ratio** | **EtOH/H_2_O (%)** | **Time** | **Orientin** | **Isoorientin** | **Isovitexin** |
| --- | --- | --- | --- | --- | --- | --- |
|  |  |  |  | mg g^-1^ | | |
| **1** | -1 | -1 | -1 | 0.70 | 0.68 | 0.17 |
| **2** | 1 | -1 | -1 | 0.23 | 0.22 | 0.06 |
| **3** | -1 | 1 | -1 | 0.54 | 0.58 | 0.14 |
| **4** | 1 | 1 | -1 | 0.11 | 0.12 | 0.03 |
| **5** | -1 | -1 | 1 | 0.68 | 0.68 | 0.18 |
| **6** | 1 | -1 | 1 | 0.34 | 0.31 | 0.09 |
| **7** | -1 | 1 | 1 | 0.55 | 0.59 | 0.15 |
| **8** | 1 | 1 | 1 | 0.17 | 0.18 | 0.05 |
| **9*** | 0 | 0 | 0 | 0.57 | 0.57 | 0.15 |

*triplicate

**Table S3:** Experimental design and responses for orientin, isoorientin and isovitexin using MAE method.

| **Experiment** | **Sample/ Solvent ratio** | **EtOH/H_2_O (%)** | **Time** | **Tempera-ture** | **Orientin** | **Isoorientin** | **Isovitexin** |
| --- | --- | --- | --- | --- | --- | --- | --- |
|  |  |  |  |  | mg g^-1^ | | |
| **1** | -1 | -1 | -1 | -1 | 0.52 | 0.70 | 0.19 |
| **2** | 1 | -1 | -1 | -1 | 0.82 | 0.78 | 0.23 |
| **3** | -1 | 1 | -1 | -1 | 0.74 | 0.74 | 0.20 |
| **4** | 1 | 1 | -1 | -1 | 0.52 | 0.67 | 0.19 |
| **5** | -1 | -1 | 1 | -1 | 0.81 | 0.85 | 0.23 |
| **6** | 1 | -1 | 1 | -1 | 0.36 | 0.36 | 0.10 |
| **7** | -1 | 1 | 1 | -1 | 0.77 | 0.77 | 0.22 |
| **8** | 1 | 1 | 1 | -1 | 0.57 | 0.70 | 0.21 |
| **9** | -1 | -1 | -1 | 1 | 0.88 | 0.82 | 0.28 |
| **10** | 1 | -1 | -1 | 1 | 0.82 | 0.94 | 0.34 |
| **11** | -1 | 1 | -1 | 1 | 0.45 | 0.67 | 0.20 |
| **12** | 1 | 1 | -1 | 1 | 0.70 | 0.79 | 0.26 |
| **13** | -1 | -1 | 1 | 1 | 0.57 | 0.67 | 0.24 |
| **14** | 1 | -1 | 1 | 1 | 0.47 | 0.64 | 0.22 |
| **15** | -1 | 1 | 1 | 1 | 0.42 | 0.60 | 0.18 |
| **16** | 1 | 1 | 1 | 1 | 0.64 | 0.70 | 0.25 |
| **17*** | 0 | 0 | 0 | 0 | 0.89 | 0.93 | 0.30 |

*quadruplicate
