# Supplementary material for: Development and application of green and sustainable analytical methods for flavonoid extraction from Passiflora waste
Source: BMC Chem. 2020 Sep 18;14(1):56. doi: 10.1186/s13065-020-00710-5 (PMC7501698; doi:10.1186/s13065-020-00710-5)
Supplement: Supplementary file 2 — Additional file 2: UV profile and QDA data of extracts from all three extraction techniques. [file 13065_2020_710_MOESM2_ESM.docx]

**Fig. S1** Normalized effects for variables 1-3 and their interactions considering HAE.

**Fig. S2** Normalized effects for variables 1-3 and their interactions considering UAE.

**Fig. S3** Normalized effects for variables 1-3 and their interactions considering MAE.
