# Supplementary material for: Development and application of green and sustainable analytical methods for flavonoid extraction from Passiflora waste
Source: BMC Chem. 2020 Sep 18;14(1):56. doi: 10.1186/s13065-020-00710-5 (PMC7501698; doi:10.1186/s13065-020-00710-5)
Supplement: Supplementary file 3 — Additional file 3: Experimental design and responses for orientin, isoorientin and isovitexin from all three extraction techniques. [file 13065_2020_710_MOESM3_ESM.docx]

Fig. S1 Molecular ion (m/z) related to peak A (Figure A1.1), identified as flavonoid orientin obtained from HAE methodology.

Fig. S2 Molecular ion (m/z) related to peak B (Figure A1.1), identified as flavonoid isoorientin obtained from HAE methodology.

Fig. S3 Molecular ion (m/z) related to peak C (Figure A1.1), identified as flavonoid isovitexin obtained from HAE methodology.

Fig. S4 Molecular ion (m/z) related to peak A (Figure A1.5), identified as flavonoid orientin obtained from UAE methodology.

Fig. S5 Molecular ion (m/z) related to peak B (Figure A1.5), identified as flavonoid isoorientin obtained from UAE methodology.

Fig. S6 Molecular ion (m/z) related to peak C (Figure A1.5), identified as flavonoid isovitexin obtained from UAE methodology.

Fig. S7 Molecular ion (m/z) related to peak A (Figure A1.9), identified as flavonoid orientin obtained from MAE methodology.

Fig.S8 Molecular ion (m/z) related to peak B (Figure A1.9), identified as flavonoid isoorientin obtained from MAE methodology.

Fig. S9 Molecular ion (m/z) related to peak C (Figure A1.9), identified as flavonoid isovitexin obtained from MAE methodology.
