# Supplementary material for: Development and application of green and sustainable analytical methods for flavonoid extraction from Passiflora waste
Source: BMC Chem. 2020 Sep 18;14(1):56. doi: 10.1186/s13065-020-00710-5 (PMC7501698; doi:10.1186/s13065-020-00710-5)
Supplement: Supplementary file 4 — Additional file 4: Results of green and sustainable factors from all three extraction techniques. [file 13065_2020_710_MOESM4_ESM.docx]

Table S1: Sustainable factors of HAE method. The values for each flavonoid were compared in the experiments and colored from green (lowest) to red (highest).

| **Analyte - Experiment** | **Extration yield (mg g^-1^)** | **Sample mass (Kg g^-1^)** | **Energy (kWh g^-1^)** | **Solvent volume (L g^-1^)** |
| --- | --- | --- | --- | --- |
| Orientin - Exp. 3 | 0,85 | 1,18 | 19,59 | 23,51 |
| Isoorientin - Exp. 3 | 1,03 | 0,97 | 16,23 | 19,48 |
| Isovitexin - Exp. 3 | 0,31 | 3,23 | 53,71 | 64,45 |
| Orientin - Exp. 4 | 0,90 | 1,11 | 9,26 | 11,12 |
| Isoorientin - Exp. 4 | 1,07 | 0,93 | 7,81 | 9,37 |
| Isovitexin - Exp. 4 | 0,33 | 3,03 | 25,26 | 30,31 |
| Orientin - Exp. 7 | 0,90 | 1,11 | 73,90 | 22,17 |
| Isoorientin - Exp. 7 | 1,07 | 0,93 | 62,31 | 18,69 |
| Isovitexin - Exp. 7 | 0,32 | 3,13 | 210,43 | 63,13 |
| Orientin - Exp. 8 | 0,94 | 1,06 | 35,46 | 10,64 |
| Isoorientin - Exp. 8 | 1,11 | 0,90 | 29,96 | 8,99 |
| Isovitexin - Exp. 8 | 0,34 | 2,94 | 97,48 | 29,24 |

Table S2: Sustainable factors of UAE method. The values for each flavonoid were compared in the experiments and colored from green (lowest) to red (highest).

| **Analyte - Experiment** | **Extration yield (mg g^-1^)** | **Sample mass (Kg g^-1^)** | **Energy (kWh g^-1^)** | **Solvent volume (L g^-1^)** |
| --- | --- | --- | --- | --- |
| Orientin - Exp. 1 | 0,70 | 1,43 | 142,86 | 28,57 |
| Isoorientin - Exp. 1 | 0,68 | 1,47 | 147,40 | 29,48 |
| Isovitexin - Exp. 1 | 0,17 | 5,88 | 579,82 | 115,96 |
| Orientin - Exp. 5 | 0,68 | 1,47 | 590,60 | 29,53 |
| Isoorientin - Exp. 5 | 0,68 | 1,47 | 589,60 | 29,48 |
| Isovitexin - Exp. 5 | 0,18 | 5,56 | 2189,05 | 109,45 |

Table S3: Sustainable factors of MAE method. The values for each flavonoid were compared in the experiments and colored from green (lowest) to red (highest).

| **Analyte - Experiment** | **Extration yield (mg g^-1^)** | **Sample mass (Kg g^-1^)** | **Energy (kWh g^-1^)** | **Solvent volume (L g^-1^)** |
| --- | --- | --- | --- | --- |
| Orientin - Exp. 10 | 0,82 | 1,22 | 116,06 | 12,19 |
| Isoorientin - Exp. 10 | 0,94 | 1,06 | 100,89 | 10,59 |
| Isovitexin - Exp. 10 | 0,34 | 2,92 | 277,87 | 29,18 |
| Orientin - Exp. 17 | 0,89 | 1,12 | 431,99 | 15,12 |
| Isoorientin - Exp. 17 | 0,93 | 1,08 | 413,56 | 14,47 |
| Isovitexin - Exp. 17 | 0,30 | 3,36 | 1292,85 | 45,25 |

Table S4: Comparison of the best values sustainable factors to each extraction method. The values for each flavonoid were compared in the experiments and colored from green (lowest) to red (highest).

|  | **Analyte - Experiment** | **Extration yield (mg g^-1^)** | **Sample mass (Kg g^-1^)** | **Energy (kWh g^-1^)** | **Solvent volume (L g^-1^)** |
| --- | --- | --- | --- | --- | --- |
| HAE | Orientin - Exp. 4 | 0,90 | 1,11 | 9,26 | 11,12 |
|  | Isoorientin - Exp. 4 | 1,07 | 0,93 | 7,81 | 9,37 |
|  | Isovitexin - Exp. 4 | 0,33 | 3,03 | 25,26 | 30,31 |
| UAE | Orientin - Exp. 1 | 0,70 | 1,43 | 142,86 | 28,57 |
|  | Isoorientin - Exp. 1 | 0,68 | 1,47 | 147,40 | 29,48 |
|  | Isovitexin - Exp. 1 | 0,17 | 5,88 | 579,82 | 115,96 |
| MAE | Orientin - Exp. 10 | 0,82 | 1,22 | 116,06 | 12,19 |
|  | Isoorientin - Exp. 10 | 0,94 | 1,06 | 100,89 | 10,59 |
|  | Isovitexin - Exp. 10 | 0,34 | 2,92 | 277,87 | 29,18 |
